# Mutants of the Zebrafish K^+^ Channel Hcn2b Exhibit Epileptic-like Behaviors

**DOI:** 10.3390/ijms222111471

**Published:** 2021-10-25

**Authors:** Roberto Rodríguez-Ortiz, Ataúlfo Matínez-Torres

**Affiliations:** 1 Cátedras CONACyT—Departamento de Neurobiología Celular y Molecular, Instituto de Neurobiología, Campus UNAM-Juriquilla, Universidad Nacional Autónoma de México, Querétaro CP 76230, Mexico; 2 Departamento de Neurobiología Celular y Molecular, Instituto de Neurobiología, Campus UNAM-Juriquilla, Universidad Nacional Autónoma de México, Querétaro CP 76230, Mexico

**Keywords:** HCN channels, epilepsy, absence seizures, zebrafish

## Abstract

Epilepsy is a chronic neurological disorder that affects 50 million people worldwide. The most common form of epilepsy is idiopathic, where most of the genetic defects of this type of epilepsy occur in ion channels. Hyperpolarization-activated cyclic nucleotide-gated (HCN) channels are activated by membrane hyperpolarization, and are mainly expressed in the heart and central and peripheral nervous systems. In humans, four HCN genes have been described, and emergent clinical data shows that dysfunctional HCN channels are involved in epilepsy. *Danio rerio* has become a versatile organism to model a wide variety of diseases. In this work, we used CRISPR/Cas9 to generate *hcn2b* mutants in zebrafish, and characterized them molecularly and behaviorally. We obtained an *hcn2b* mutant allele with an 89 bp deletion that produced a premature stop codon. The mutant exhibited a high mortality rate in its life span, probably due to its sudden death. We did not detect heart malformations or important heart rate alterations. Absence seizures and moderate seizures were observed in response to light. These seizures rarely caused instant death. The results show that mutations in the Hcn2b channel are involved in epilepsy and provide evidence of the advantages of zebrafish to further our understanding of the pathogenesis of epilepsy.

## 1. Introduction

Epilepsy is a brain disorder characterized by recurrent spontaneous seizures due to abnormal excessive electrical discharges of brain neurons. The disease affects about 1% of the world population, and is a devastating neurological disorder (www.who.int, 15 August 2021). Genetic factors play a crucial role in the origin of epilepsy, with reported mutations in many genes associated with neural function, among them numerous genes encoding voltage-gated ion channels [1,2]. It is not surprising that many currently available medications for this disease restore the balance between excitatory and inhibitory neurotransmission by selectively targeting ion channels.

Hyperpolarization-activated cyclic nucleotide-gated (HCN) channels are the molecular correspondents of the heart’s funny currents, or *I_f_*, which are responsible for pacemaker activity [3,4,5]. HCNs are also widely expressed in the central and peripheral nervous systems where they have an active role in neuronal excitability, rhythmic neuronal activity, dendritic integration, and synaptic transmission, although novel functions are emerging for this family of channels [6]. There are four mammalian HCN genes termed HCN1–4, that share a high sequence identity but exhibit molecular and functional heterogeneity [7]. HCN1, HCN2 and HCN4 show a differential and complementary expression pattern in the neurons of the central nervous system (CNS), where they assemble as homo- or heteromultimers [8]. 

HCN channels belong to the superfamily of six transmembrane segment channels, and are structurally related to cyclic nucleotide-gated (CNG) channels and voltage-dependent K^+^ (Kv) channels. HCNs have a selectivity filter typical of K^+^ channels, but they allow the passing of both K^+^ and Na^+^ [9,10]. Since their discovery, and due to their fundamental neurophysiological roles, HCN channels have been considered excellent epilepsy candidate genes, and emergent clinical data shows that dysfunctional HCN channels are indeed involved in epilepsy [11]. Thus, multiple lines of evidence implicate an association between HCN gene mutations and human hereditary epilepsy. For example, HCN1 and HCN2 functional gene variants that result in an alteration in the amino acids critical to conferring the electrophysiological characteristics of the channel are associated with idiopathic generalized epilepsy [12]. The importance of HCN malfunction in epilepsy has also been highlighted by the identification of a deletion of three consecutive prolines in HCN2 in patients that have generalized epilepsy with febrile seizures [13]. 

There is also evidence of a loss-of-function point mutation in HCN2 that contributes to generalized epilepsy in humans [14]. In this study, a screening in families with epilepsy identified a recessive point mutation in HCN2 [14]. More evidence of the association of HCN2 with epilepsy was provided by the identification of a heterozygous missense mutation (S126L) found in children with febrile seizures, which suggests that the mutation may contribute to neuronal hyperexcitability [15].

Animal models have provided more evidence of the association of mutations in HCN2 with neurophysiological alterations. An HCN2-deficient mouse exhibited increased thalamic bursts and absence epilepsy, which is a sudden loss of consciousness and behavioral arrest (as reviewed in Barone et al., 2020 [16]). In another case, an HCN2-null mouse presented reduced locomotor activity, ataxia, and cardiac sinus dysrhythmia [17]. 

As illustrated above, mouse models have proved important for studying epilepsy related to HCN2 mutations. However, there is also a need for in vivo disease models that are amenable to high-throughput small molecule screens. Zebrafish (*Danio rerio*) seizure models have become significant contributors in epilepsy research. The first zebrafish epilepsy model was chemically induced by pentylenetetrazole (PTZ), and was fully characterized (behavior, gene expression, electrophysiology, and pharmacology) [18]. This work is still a referent in zebrafish epilepsy models. One of the most studied epilepsy models in zebrafish is related to monogenic mutations in Nav1.1 (*scn1Lab*), a voltage-gated sodium channel that causes Dravet syndrome [19,20]. When CRISPR/Cas9 was introduced in zebrafish [21,22], this technology was quickly applied for the study of brain organization, function, and connectivity, as well as associated diseases such as epilepsy [23]. For example, a monogenetic epilepsy has been described using CRISPR/Cas9 to target syntaxin-binding protein 1 gene homologs in zebrafish, Stxbp1a and Stxb1b [24]. Interestingly, while Stxb1a mutants exhibited a profound lack of movement, low electrical brain activity, low heart rate, decreased glucose and mitochondrial metabolism, and early lethality, the *stxb1b* homozygous mutant allele showed spontaneous seizures and a reduced locomotor activity response to a movement-inducing “dark-flash” visual stimulus, despite showing a normal metabolism, heart rate, survival, and baseline locomotor activity [24]. Another example in the study of epilepsy is the zebrafish *aldh7a1* null mutant, which recapitulates the characteristics of pyridoxine-dependent epilepsy, caused by variants of the gene ALDH7A1. The homozygous mutants showed a spontaneous, quick increase in locomotion and a rapid, circling swimming behavior followed by seizure-like behaviors that resulted in death shortly after a seizure [25,26].

To shed some light on the role of HCN channels in epilepsy, in this work we generated a null mutant of the gene *hcn2b* of zebrafish using CRISPR/Cas9 technology, and characterized the behavioral consequences of the mutation.

## 2. Results

### 2.1. Generation and Characterization of the Hcn2b-KO Zebrafish Line

**Figure 1:**
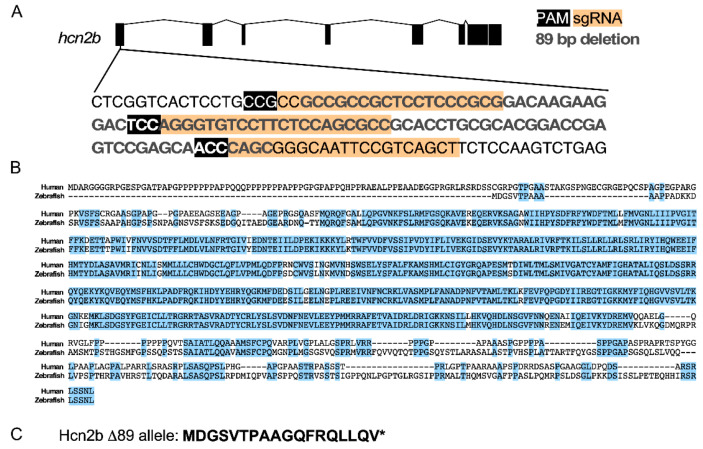
Zebrafish *hcn2b* gene, Human HCN2 and zebrafish Hcn2b protein alignment and the Hcn2b Δ89 allele. (**A**) Exon-intron organization of the zebrafish *hcn2b*. The three sgRNA Cas9-targeted are in the first exon. Bold grey letters represent the 89 pb deletion of the *hcn2b*^−/−^ allele. (**B**) Alignment of predicted primary sequences of human HCN2 and zebrafish Hcn2b. Similar amino acids are highlighted in blue. (**C**) Primary sequence of the 18 amino acids predicted in the *hcn2b*^−/−^ allele.

**Figure 2:**
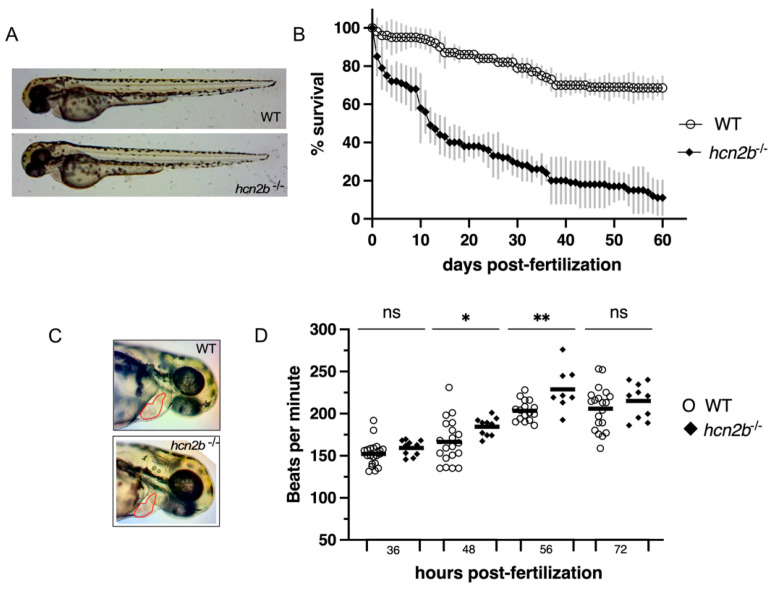
Characterization of the *hcn2b*^−/−^ mutant. (**A**) Compared morphology between WT and *hcn2b*^−/−^ zebrafish mutant at 48 h post-fertilization (hpf): there are no differences in length and pigmentation. (**B**) Homozygous *hcn2b* mutants showed a higher mortality rate than controls (4–16% vs. 60–80%). (**C**) No evident defects were detected during early heart development. Both heart morphology and volume were similar in WT and *hcn2b*^−/−^ (**D**). Heart rate showed statistically significant differences between WT and *hcn2b*^−/−^ larvae at 48- and 56-h post-fertilization. Asterisk indicate statistical significance compared to the control, *p* < 0.05 (*); *p* < 0.005 (**).

The *hcn2b* gene displays eight exons (Figure 1A) and encodes a predicted protein of 979 amino acids, containing the typical characteristics of the HCN channel family: six transmembrane domains, a cyclic nucleotide binding domain, and a C-linker (Figure S1). CLUSTAL alignment showed that the zebrafish Hcn2b protein (Gene ID: 528516153) shares an amino acid sequence identity of 72% with the human HCN2 protein (879 amino acids) (GI: 156071470) (Figure 1B). Therefore, mutations in *hcn2b* that disrupt the function of the channel were likely to affect ion currents. To evaluate the Hcn2b channel’s contribution to producing an epileptic phenotype in zebrafish, we generated mutations using the CRISPR/Cas9 system, combining three sgRNAs to enhance the probability of inducing a frameshift mutation in F0 animals, also called crispants. We chose three close target sites in exon 1 of the *hcn2b*, separated only by 13 and 29 bp between each sgRNA (Figure 1A). Because of the high rate of mortality in crispants (see below, Figure 2B), we decided to grow the survivors to adulthood to perform heterozygous mating to produce F1 descendants at the expected Mendelian ratio. An 89 bp deletion within the first exon of *hcn2b* that introduced a premature stop codon at the amino acid position 19 was confirmed by the screening of F1 descendants by PCR amplification and sequencing (Figure 1C). Then, we selected the parents that displayed the 89 bp deletion to establish the F2 homozygous *hcn2b*^Δ^^89/Δ^^89^ mutant zebrafish, hereafter referred to as *hcn2b*^−/−^. We noticed that the 89 bp deletion was the result of a canonical cut made by Cas9, located between the third and fourth nucleotide from the PAM sequence at both of the sgRNA ends, followed by a DNA double-strand break repair. We maintained hetero- and homozygous *hcn2b* adult zebrafish mutants separately for outcrossing. 

### 2.2. Phenotype and Survival Rate

Both, heterozygous *hcn2b^+/−^* and homozygous *hcn2b*^−/−^ mutant fish have normal gross morphology (Figure 2A) and are capable of growing and mating. 

Homozygous *hcn2b*^−/−^ mutant larvae showed a higher mortality rate during their life span. We followed three different groups for 60 days (N = 3, n = 210), in which about 30% of the homozygous *hcn2b*^−/−^ mutant larvae had died by two days post-fertilization (dpf), in contrast to the 10% mortality observed in control fish. Furthermore, at 10 dpf, there was a notorious mortality increase in the mutant larvae that leveled off at 60 dpf for a final 10–25% survival rate; in comparison, around 80% of the control fish survived (Figure 2B). Since *hcn2b* is strongly expressed in the heart and CNS, the higher mortality in homozygous *hcn2b*^−/−^ larvae could be explained by a sudden death induced by heart malfunction, after a severe epileptic episode, or a combination of both. We rarely observed spontaneous epileptic episodes when we cleaned, fed, or manipulated the mutant larvae, although we incidentally identified hyperactivity in some samples. In addition, we observed “paralyzed” larvae at the bottom of the tanks on several occasions. The behaviors observed during this period were later corroborated by the following behavior assays. 

### 2.3. Heart Rate and Cardiac Morphology

Because of the implication of the HCN2 in pacemaking function in the heart, we evaluated the heart rate and morphology of the *hcn2b* mutant zebrafish. Homozygous *hcn2b^−^*^/*−*^ mutant zebrafish had normal heart morphology (Figure 2C). No evident malformations were detected, and the area of the heart was similar between controls and *hcn2b* mutants. In order to determine the heart rate, we tested the fish at 36-, 48-, 56- and 72-h post-fertilization (hpf). The *hcn2b*^−/−^ mutant larvae (n = 15) showed a statistically significant increment in the mean heart rate compared to sibling controls (n = 20) at 48 and 56 hpf (*p* = 0.0441 and *p* = 0.0029) in a two-tailed *t*-test. The heart rates at 36 hpf and 72 hpf showed no statistical differences (Figure 2D). 

### 2.4. Larval and Adult Behavioral Analysis

#### 2.4.1. Spontaneous Tail Movement

**Figure 3:**
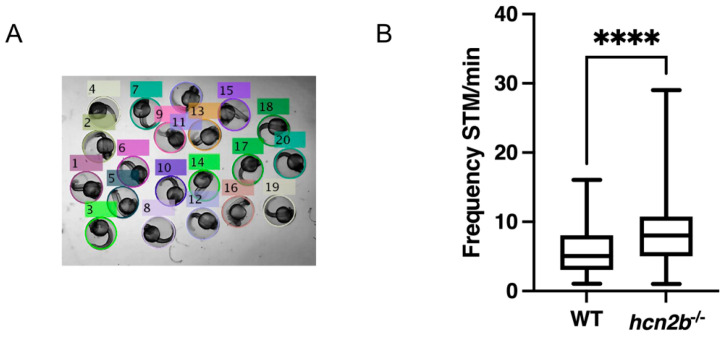
Spontaneous tail movement. (**A**) Twenty-four hours post-fertilization zebrafish larvae in a glass slide; tagged and numbered by MATLAB application ZebraSTM [27]. (**B)** Frequency of spontaneous tail movements (STM) per one minute of WT versus *hcn2b*^−/−^ zebrafish mutant. Homozygous *hcn2b* mutants showed 1.5 times more tail movements compared to WT. Asterisk indicate statistical significance compared to the control, *p* < 0.0001 (****).

Motor activity can be evaluated by observing the spontaneous tail coiling as early as 17 hpf [27]. One-minute video recordings of groups of larvae at 24 hpf were obtained and processed in the open-source MATLAB application ZebraSTM [28]. The mean of the spontaneous tail coiling of the control group (mean = 5.875 coils/min) was statistically lower compared with the *hcn2b*^−/−^ mutant (mean = 9.167 coils/min) (n = 72, *p* ≤ 0.0001 in a two-tailed *t*-test). Thus, the *hcn2b*^−/−^ zebrafish mutant had 1.5 times more spontaneous tail movements at this age (Figure 3). 

#### 2.4.2. Dark-Light Transitions

**Figure 4:**
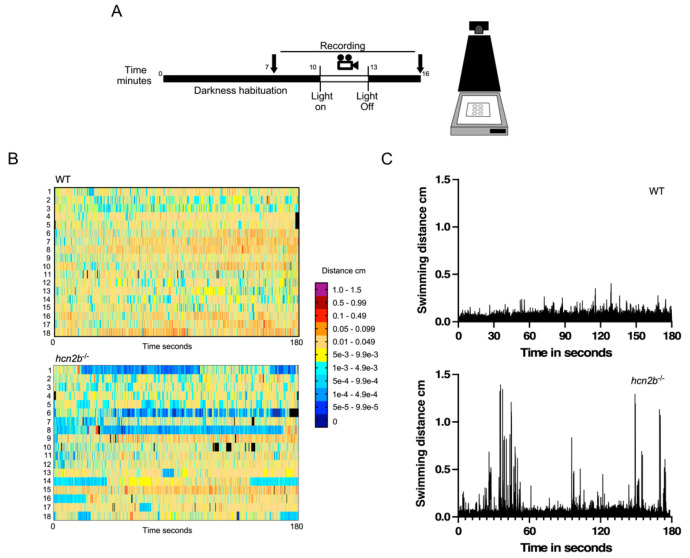
Motility during dark-light-dark transitions. (**A**) Video recording protocol. At seven days post-fertilization, larvae were placed in 23 mm diameter wells and covered from light for ten minutes for habituation to darkness, then exposed to three minutes of continuous light and, finally, three minutes of darkness. (**B**) Heatmap of the swimming behavior of WT and *hcn2b*^−/−^. 18 WT and 18 *hcn2b* mutants are shown. Low activity periods that last over time (in blue) can be interpreted as absence periods (larvae 1, 6 and 8). The high activity in red and purple are fast in a short period in time (see *hcn2b*^−/−^ (larvae 1, 8, 15 and 18). (**C**) Swimming distances of 18 WT and 18 *hcn2b*^−/−^ mutants in the light period. The *hcn2b*^−/−^ mutants displayed brief peaks as indication of fast hyperactivity episodes.

To examine the changes in locomotor activity and responses to visual light stimulation, we quantified the movement of individual zebrafish larvae at 7 dpf during dark-light-dark transitions (Figure 4A). During an initial and final dark period, neither the controls nor the *hcn2b*^−/−^ mutants displayed behavioral differences. After a sudden light exposure, we observed evident behavior differences between the two groups tested. We observed that several *hcn2b*^−/−^ mutant larvae paralyzed or reduced their swimming as a response to the light, which we interpreted as an absence seizure (Figure 4B, Video S1). During the test, we also noticed fast episodes of hyperactivity that became evident when we analyzed the swimming distances during the three minutes of light exposure, in which we observed peaks of activity beyond the threshold (Figure 4C). We evaluated the conditions of the larvae one hour after the test was completed and did not detect any death caused by hyperactivity or absence epilepsy induced by this assay.

#### 2.4.3. Light Flashes

**Figure 5:**
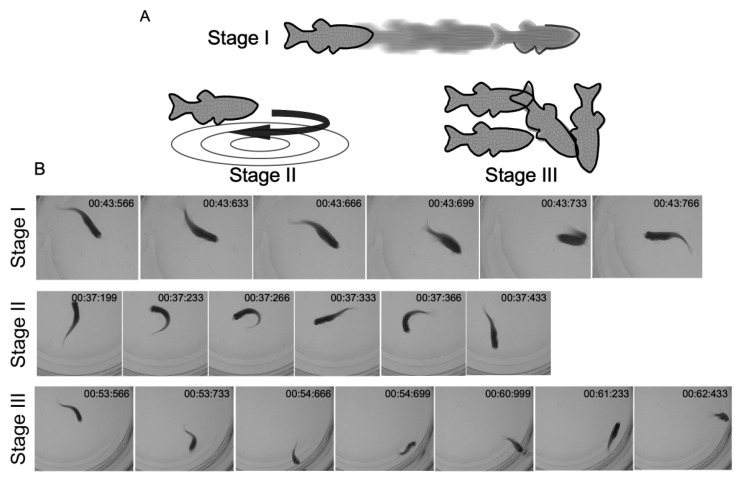
Seizures induced by flashlights in the *hcn2b*^−/−^ mutant. During dark-light transitions, about 7% of adult *hcn2b*^−/−^ mutants exhibited behavioral seizure activity. (**A**) First, fish increased their swimming activity (Stage I); then, they showed a rapid “whirlpool-like” circling swimming behavior (Stage II); finally, they exhibited a series of brief clonus-like convulsions that led to a loss of posture and immobility (Stage III). (**B**) Snap shot sequences of each stage of the seizures caused by flashlight in the *hcn2b*^−/−^ mutant zebrafish. Images taken from Video S2.

Young adult fish from five months post-fertilization were exposed to a sudden light flash to test for photosensitivity in *hcn2b* mutants. About 7% of the mutants showed either absence epilepsy or seizures (n = 70). Absence epilepsy episodes lasted up to one minute and the locomotor recovery was instantaneous. Seizures appeared immediately upon turning on the lights and increased progressively following the three stages described by [26]: first, an increment in swimming activity; then, a rapid circling swimming behavior; and, finally, brief clonus-like convulsions that led to a loss of normal body posture and immobility (Figure 5, Video S2). These behaviors could be repeated more than once, and were separated by short periods of recovery before returning to the normal posture. We followed the fish for one hour after the test was completed and we did not detect any death caused by this assay. 

#### 2.4.4. Novel Tank Diving Assay

**Figure 6:**
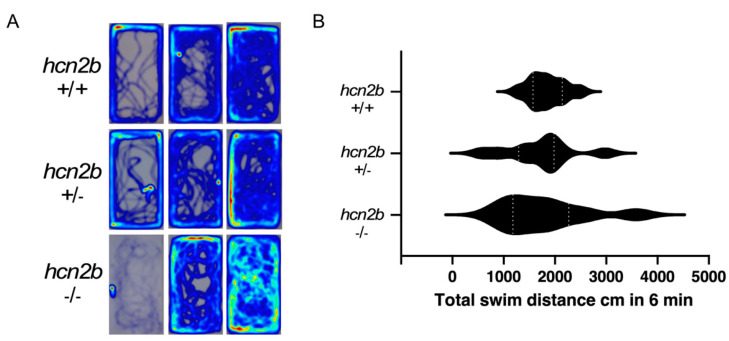
Novel tank diving assay. (**A**) Heat maps of three samples (low, medium and high activity) of the WT, hetero- and homozygous *hcn2b* zebrafish mutants in the swimming performance for six minutes in the novel tank. (**B**) Total swimming distances of the WT, hetero- and homozygous *hcn2b* zebrafish mutants. No statistical differences were observed between groups but *hcn2b*^−/−^ displayed heterogeneous values that corresponded with moments of hyperactivity and immobility. The heat maps and swimming distances were analyzed using EthoVisionXT (Noldus IT, Wageningen, The Netherlands).

This assay is commonly used to measure anxiety and is useful to examine thigmotaxis, a well-known behavior observed when animals are in a novel environment. Here we evaluated two parameters: 1) total distance traveled throughout the entire tank and 2) total time spent in different zones. As expected, at the beginning of the test, the control fish swam cautiously to explore the edges of the tank and progressively started crossing the midline, which is usually interpreted as a reduction of anxiety after habituation (Figure 6, Video S3). Homozygous *hcn2b^−^*^/*−*^ mutant zebrafish showed a diverse behavior. For instance, in some cases, the anxiety caused by the new environment induced absence epilepsy, which was evident because after transferring the fish to the tank, the fish paralyzed or dramatically reduced swimming activity; consequently, the distance traveled was shorter compared to controls. In other cases, the anxiety induced hyperactivity in the fish, which started swimming frenetically, covering all the zones of the tank. Yet in other cases, a period of absence or low activity ensued from a period of hyperactivity. Sample trajectories can be observed in the heat map of Figure 6. Although the mean values of the traveled distance are not statistically significant among the three groups (*p* = 0.7365, Kruskal–Wallis test), the heterozygous *hcn2b* mutants showed an intermediate behavior, but mostly hyperactivity (Figure 6, Video S3).

## 3. Discussion

Here, we report the generation of a mutant of the *hcn2b* gene of zebrafish to explore its effects on neurological disorders, such as epilepsy, and cardiac deficiencies. *Danio rerio*, a teleost fish, may have a second copy of HCN2 since its genome was duplicated during evolution, which often results in two co-ortholog genes. Even though the zebrafish information network located *hcn2a* in chromosome 2, the lack of additional information made the presence of a possible *hcn2a* gene debatable. Of course, this does not discard their presence in the zebrafish genome, however our experimental evidence provides functional information of the impact of the absence of *hcn2b*. We demonstrated a range of embryonic, larval, and adult phenotypes relevant to Hcn2b missfunction, including signs observed in epileptic patients (e.g., absence crisis, seizures, and motor deficits). These characteristics support that zebrafish mutants do indeed recapitulate many features of HCN-related diseases. Therefore, this zebrafish model would be a useful tool for studying epilepsy. Since seizures and absence crises are common symptoms of the human disease, Hcn2b-deficient zebrafish mutants may be useful for studying these events.

The homozygous mutation of *hcn2b* generated here, *hcn2b*^−/−^, does not cause apparent morphological changes, since the animals presented normal length, mobility, and pigmentation. Although we did not monitor the weight nor the length of adult fish, we did not observe impaired growth with respect to the WT control as reported in mice [29]. However, during their lifetime, the mutants exhibited a higher rate of mortality. At this point, the causes of death are merely speculative, and may be related to a cardiac malfunction combined with photosensitivity that led to epileptic episodes including absence and seizures. 

HCN channels are the molecular components of the heart pacemaker current, a current activated by hyperpolarization that triggers the sinus node function to generate heart impulses. HCN2 and HCN4 are widely expressed in the sinoatrial node, although the absence of HCN2 in mice did not affect the mean heart rate during spontaneous movements [14,30,31]. However, the intervals between successive heart beats varied widely at rest due to a dysfunction of the sinoatrial node. Thus, it seems that HCN2 has a role in the pacemaking activity under non-stimulated conditions [17]. In *hcn2b*^−/−^ mutant zebrafish, we identified a slight increment in the heart rate at 48 and 56 hpf, reflecting a shorter time interval between successive heart beats, resembling that of the viable HCN2-deficient mice [17]. It would be interesting to monitor the heart‘s activity during the zebrafish spontaneous movement in larvae or adult zebrafish to establish if there is an association between the sudden death and heart malfunction. 

Early development of locomotion in zebrafish can be evaluated by spontaneous tail movement as early as 17 hpf [27,32,33]. Motor function development in fish is evident by the appearance of contraction patterns of the axial musculature necessary for swimming. In our case, we wanted to examine if the lack of the Hcn2b channel produced alterations in the early development of the neural system that affected such contraction patterns. The differences in the number of events between the Hcn2b mutant and Wild-type (WT) after 24 hpf were remarkable: 5.875 coils/min vs. 9.167 coils/min, respectively. These spontaneous contractions are likely related to the eventual hatching of the embryo [34]; thus, it was unexpected to observe that the mutant fish, which have about 1.5 times more contractions, hatched at the same time (around 3–4 dpf) compared with the WT controls, as well as the hatching times reported elsewhere [28,32,33].

Epilepsy-related behaviors are usually composed of subtle changes in locomotion, including atonic and absence seizures, tremor, and myoclonic twitches, among others. In contrast to the behavioral changes of the HCN2-deficient mice, which reduced their locomotor activity and displayed absence epilepsy [17], we detected hyperactivity, seizures, and absence epilepsy in the *hcn2b* mutant zebrafish. These opposite behaviors may be explained by physiological differences between both species. However, the nocturnal habits of mice and the diurnal habits of zebrafish may be related, as suggested by the photosensitivity exhibited by the mutant fish.

We also observed that the hyperactive phenotype exhibited when the lights were turned on in the early morning was progressive in the *hcn2b* mutants. First, it was suggested by sporadic abnormal movements in the *hcn2b* crispant larvae, but later it was quite evident in the heterozygous and homozygous fish. During the evaluation of the locomotor activity of the larvae in response to dark-light transitions, we clearly observed two different responses in the *hcn2b*^−/−^ mutants in the dark-to-light transition: (1) around 30% of the *hcn2b*^−/−^ mutant larvae shown periods of absence, whereas (2) around 60% showed a peak of hyperactivity throughout the light stage. Wild-type larvae maintained their locomotor activity during the three-minute light period, as previously reported [35,36]. Epileptic and absence seizures can be a consequence of photosensitivity in the *hcn2b*^−/−^ mutant larvae, in response to the stress and anxiety induced by the sudden change of environmental light.

A recent study found that photosensitivity contributes to generalized epilepsy in variants of gain-of-function heterozygous HCN2 patients [37]. This prompted us to test whether *hcn2b* mutants are photosensitive by exposing young adult fish to a sudden flash light. In this condition, about 7% of the mutants showed either absence epilepsy or seizures. Absence epilepsy episodes were less common than seizures and lasted up to one minute followed by a rapid locomotor recovery. Seizures appeared right when the lights were turned on after the period of dark habituation, then they increased progressively and showed distinct phases: (1) an increment in swimming activity; (2) a rapid, circling swimming behavior; (3) brief clonus-like convulsions leading to a loss of posture; and (4) periods of immobility. We observed this behavioral pattern repeatedly, divided by brief periods of recovery. These behaviors were rarely observed spontaneously at any age.

The role of the stress response in the onset and progression of epilepsy is well recognized and related to the incidence and severity of the disease [38,39]. Therefore, we examined the response of *hcn2b* mutants to a widely-used stressor in animal models: the challenge of a new and unknown environment. Using the novel tank test, we assessed if anxiety could trigger a reaction to induce changes in locomotor activity or zone preferences. Although we did not detect evident differences in the total distance traveled between *hcn2b* mutants and WT controls, we observed two intriguingly different and opposite behaviors: some individuals showed hyperactivity, while others showed absence epilepsy. Diverse studies in murine models have suggested that exposure to acute stressors may protect against seizures in contrast to chronic stressors, which increases seizure risk and frequency [40,41]. The rapid response to a stressor activates the sympathetic-adrenomedullary system which, in turn, activates the sympathetic nervous system. We do not yet know the expression pattern of the Hcn2b zebrafish, but we suspect it is widely distributed in the peripheral and central nervous system, like in mammals. Thus, the diverse responses observed in the *hcn2b* mutants may arise from a malfunction of these systems, which may give rise to the diversity of behaviors.

The physiological understanding of the diversity of the responses of the *hcn2b* mutant zebrafish will require a detailed study of this new epilepsy model. The easy handling and maintenance of the fish will be helpful to explore the role of HCNs in other fields of current interest, such as those of pain and depression [42,43,44,45]. Our results show that mutations in the Hcn2b channel are clearly involved in epilepsy, which may be of pathogenic and pharmacological relevance, and also can be useful for the study of the role of HCN channel function in other neurological diseases.

## 4. Materials and Methods

### 4.1. Animals

Zebrafish of the AB-Tu-WIK hybrid line were maintained in standard conditions, and all experimental procedures were performed according to the protocol approved by the Animal Care and Use Committee of the Institute of Neurobiology, National Autonomous University of Mexico (Protocol number 95A). Natural spawning was used to collect embryos which were raised at 28oC in 100 mm plates with Blue E3 medium (5 mM NaCl, 0.17 mM KCl, 0.33 mM CaCl_2_, 0.33 mM MgSO4 and 0.0001% methylene blue, pH 7.2). Embryo plates were cleared of debris and dead fish daily and Blue E3 medium was added to the plates as needed. Fish from 5 up to 60 dpf were maintained in 3 L tanks with fish water (Marine salts, Instant Ocean, Blacksburg, VA, USA) at pH 7.5 and 500-750 us conductivity. Adult fish were maintained in polycarbonate tanks in a system with re-circularized fish water.

### 4.2. Generation of Zebrafish hcn2b Mutations by CRISPR/Cas9

Three sgRNA sequences directed to *hcn2b* were selected using the CRISPR design tool CRISPRscan [46]. All three sgRNA target sites are located in exon 1 of *hcn2b*: a: CCGCCGCTCCTCCCGCG, b: GGGTGTCCTTCTCCAGCGCC, and c: AGCGGGCAATTCCGTCAGCT. The sgRNA was synthesized using the protocol described by Vejnar et al. (2016). Cas9 mRNA was synthesized using *Xba*I linearized pT3TSnCas9n plasmid as a template for in vitro transcription [47]. An RNA mixture of sgRNA (25 ng/uL) and Cas9 mRNA (300 ng/uL) was microinjected into single-cell stage zebrafish embryos. After 48–72 hpf, 10 injected embryos were selected to extract genomic DNA by Hot-Shot [48] and PCR was performed to test the CRISPR/Cas9 system efficiency. We designed two primers (CTCCAGCATGGACGGCTCGG and CTGGCCATCCTCAGACTTGG, forward and reverse respectively) to characterized the mutations which amplified a 427 bp fragment in the WT.

### 4.3. Protein Alignment Analysis

HCN2 protein sequence from human (Gene ID: 156071470) and Hcn2b from zebrafish (Gene ID: 528516153) were obtained from Ensembl (www.ensembl.org, 15 August 2021), with accession numbers ENST00000251287.3 and ENSDART00000136390.2, respectively. Protein alignment was done using MEGA X.

### 4.4. Heart Rate

We generated an easy-to-use MATLAB script to determine the heart rate from 1 min larvae videos. For video recordings, we used a Celestron camera (Torrance, CA, USA) coupled to a stereoscope. Larvae from 36 hpf to 72 hpf were video recorded individually for 1 min in a lateral position when the heart was evidently beating. Then, we loaded the videos in the MATLAB script (available upon request) and selected the region of interest to determine the number of beats and, after an automatic correction, we obtained the heart rate (beats/minute).

### 4.5. Larval and Adult Behavioral Analysis

#### 4.5.1. Spontaneous Tail Movement

Motor activity can be assessed by spontaneous tail coiling as early as 17 hpf. To perform the tail movement assay, we video recorded a group of 15–25 larvae for 1 min at 24 hpf in the same plate immersed in blue E3 medium. To habituate the larvae to the stereoscope light, we placed the plate under this condition 2 min before video capture. The video was processed in the open-source MATLAB application ZebraSTM [28] and the spontaneous tail movements were quantified and plotted for comparison.

#### 4.5.2. Dark-Light Transitions

To evaluate the early response of larvae to dark-light transitions, we designed a quick test as follows. We placed individual 7 dpf larvae into a 12-well plate filled with Blue E3 water. The plate was set above a light chamber and covered with black conical plastic with a video camera at the top. We habituated the larvae to darkness for 10 min. At minute 7 of this period, we began the recording in the dark before turning the lights on and video recording for 3 more minutes. Finally, we turned the lights off to record the larvae for 3 more minutes. The trajectories were analyzed using the software EthoVisionXT (Noldus IT, Wageningen, The Netherlands).

#### 4.5.3. Light Flashes

Adult fish were exposed to a light flash in order to test photosensitivity. Individual adult fish from 5 months post-fertilization were placed in 250 mL beakers filled with 150 mL of fish water. The beakers were placed above a light chamber and covered with a black conical plastic coupled to a video camera. We habituated the fish to the dark for 10 min. At minute 7 of this period, we started recording in the dark before turning the lights on and recorded 3 more minutes. Finally, the lights were turned off and the recording continued for 3 more minutes.

#### 4.5.4. Novel Tank Diving Assay

This is a commonly used test for anxiety. Prior to performing the experiment, we standardized the manipulation of the larvae, including the time spent in netting to avoid stress and maintain the same fish water conditions (pH, conductivity, temperature). Individual fish were carefully transferred from home tanks to a 250 mL beaker filled with fish water from the home tank. After 10 min of habituation, fish were transferred to a 1 L examination tank filled with 125 mL of fresh water. A 6 min video was recorded from the top of the tank, and the trajectories were analyzed using EthoVisionXT (Noldus IT, Wageningen, The Netherlands).

### 4.6. Software and Statistical Analyses

All statistical analyses were performed using Prism9 (GraphPad, San Diego, CA, USA). Heart rate analysis was performed in MATLAB R2015a (MathWorks, Natick, MA, USA). Figures were prepared with Canvas X Draw version 7.0.2 (Boston, MA, USA) and the videos were edited in Movavi Slideshow Maker 5 version 5.4.0 (Wildwood, MO, USA).

## Supplementary Materials

The following are available online at https://drive.google.com/drive/folders/11Y7NfiXzNZn00SnDtSwuGEKDp8Qy5nHS.

## References

[1] Deng H., Xiu X., Song Z. (2014). The Molecular Biology of Genetic-Based Epilepsies. Mol. Neurobiol..

[2] Perucca P., Bahlo M., Berkovic S.F. (2020). The Genetics of Epilepsy. Annu. Rev. Genom. Hum. Genet..

[3] Ludwig A., Zong X., Jeglitsch M., Hofmann F., Biel M. (1998). A Family of Hyperpolarization-Activated Mammalian Cation Channels. Nature.

[4] Santoro B., Grant S.G.N., Bartsch D., Kandel E.R. (1997). Interactive Cloning with the SH3 Domain of N-Src Identifies a New Brain Specific Ion Channel Protein, with Homology to Eag and Cyclic Nucleotide-Gated Channels. Proc. Natl. Acad. Sci. USA.

[5] Santoro B., Liu D.T., Yao H., Bartsch D., Kandel E.R., Siegelbaum S.A., Tibbs G.R. (1998). Identification of a Gene Encoding a Hyperpolarization-Activated Pacemaker Channel of Brain. Cell.

[6] Santoro B., Tibbs G.R. (1999). The HCN Gene Family: Molecular Basis of the Hyperpolarization-Activated Pacemaker Channels. Ann. N. Y. Acad. Sci..

[7] Santoro B., Chen S., Lüthi A., Pavlidis P., Shumyatsky G.P., Tibbs G.R., Siegelbaum S.A. (2000). Molecular and Functional Heterogeneity of Hyperpolarization-Activated Pacemaker Channels in the Mouse CNS. J. Neurosci..

[8] Brennan G.P., Baram T.Z., Poolos N.P. (2016). Hyperpolarization-Activated Cyclic Nucleotide-Gated (HCN) Channels in Epilepsy. Cold Spring Harb. Perspect. Med..

[9] Lee C.-H., MacKinnon R. (2017). Structures of the Human HCN1 Hyperpolarization-Activated Channel. Cell.

[10] Lee C.-H., MacKinnon R. (2019). Voltage Sensor Movements during Hyperpolarization in the HCN Channel. Cell.

[11] Chang X., Wang J., Jiang H., Shi L., Xie J. (2019). Hyperpolarization-Activated Cyclic Nucleotide-Gated Channels: An Emerging Role in Neurodegenerative Diseases. Front. Mol. Neurosci..

[12] Tang B., Sander T., Craven K.B., Hempelmann A., Escayg A. (2008). Mutation Analysis of the Hyperpolarization-Activated Cyclic Nucleotide-Gated Channels HCN1 and HCN2 in Idiopathic Generalized Epilepsy. Neurobiol. Dis..

[13] Dibbens L.M., Reid C.A., Hodgson B., Thomas E.A., Phillips A.M., Gazina E., Cromer B.A., Clarke A.L., Baram T.Z., Scheffer I.E. (2010). Augmented Currents of an HCN2 Variant in Patients with Febrile Seizure Syndromes. Ann. Neurol..

[14] DiFrancesco J.C., Barbuti A., Milanesi R., Coco S., Bucchi A., Bottelli G., Ferrarese C., Franceschetti S., Terragni B., Baruscotti M. (2011). Recessive Loss-of-Function Mutation in the Pacemaker HCN2 Channel Causing Increased Neuronal Excitability in a Patient with Idiopathic Generalized Epilepsy. J. Neurosci..

[15] Nakamura Y., Shi X., Numata T., Mori Y., Inoue R., Lossin C., Baram T.Z., Hirose S. (2013). Novel HCN2 Mutation Contributes to Febrile Seizures by Shifting the Channel’s Kinetics in a Temperature-Dependent Manner. PLoS ONE.

[16] Barone V., van Putten M.J.A.M., Visser G.H. (2020). Absence Epilepsy: Characteristics, Pathophysiology, Attention Impairments, and the Related Risk of Accidents. A Narrative Review. Epilepsy Behav..

[17] Ludwig A. (2003). Absence Epilepsy and Sinus Dysrhythmia in Mice Lacking the Pacemaker Channel HCN2. EMBO J..

[18] Baraban S.C., Taylor M.R., Castro P.A., Baier H. (2005). Pentylenetetrazole Induced Changes in Zebrafish Behavior, Neural Activity and c-Fos Expression. Neuroscience.

[19] Baraban S.C., Dinday M.T., Hortopan G.A. (2013). Drug Screening in Scn1a Zebrafish Mutant Identifies Clemizole as a Potential Dravet Syndrome Treatment. Nat. Commun..

[20] Grone B.P., Qu T., Baraban S.C. (2017). Behavioral Comorbidities and Drug Treatments in a Zebrafish *Scn1lab* Model of Dravet Syndrome. ENeuro.

[21] Chang N., Sun C., Gao L., Zhu D., Xu X., Zhu X., Xiong J.-W., Xi J.J. (2013). Genome Editing with RNA-Guided Cas9 Nuclease in Zebrafish Embryos. Cell Res..

[22] Hwang W.Y., Fu Y., Reyon D., Maeder M.L., Tsai S.Q., Sander J.D., Peterson R.T., Yeh J.-R.J., Joung J.K. (2013). Efficient Genome Editing in Zebrafish Using a CRISPR-Cas System. Nat. Biotechnol..

[23] Espino-Saldaña A.E., Rodríguez-Ortiz R., Pereida-Jaramillo E., Martínez-Torres A. (2020). Modeling Neuronal Diseases in Zebrafish in the Era of CRISPR. Curr. Neuropharmacol..

[24] Grone B.P., Marchese M., Hamling K.R., Kumar M.G., Krasniak C.S., Sicca F., Santorelli F.M., Patel M., Baraban S.C. (2016). Epilepsy, Behavioral Abnormalities, and Physiological Comorbidities in Syntaxin-Binding Protein 1 (STXBP1) Mutant Zebrafish. PLoS ONE.

[25] Pena I.A., Roussel Y., Daniel K., Mongeon K., Johnstone D., Weinschutz Mendes H., Bosma M., Saxena V., Lepage N., Chakraborty P. (2017). Pyridoxine-Dependent Epilepsy in Zebrafish Caused by Aldh7a1 Deficiency. Genetics.

[26] Zabinyakov N., Bullivant G., Cao F., Fernandez Ojeda M., Jia Z.P., Wen X.-Y., Dowling J.J., Salomons G.S., Mercimek-Andrews S. (2017). Characterization of the First Knock-out Aldh7a1 Zebrafish Model for Pyridoxine-Dependent Epilepsy Using CRISPR-Cas9 Technology. PLoS ONE.

[27] Saint-Amant L., Drapeau P. (1998). Time Course of the Development of Motor Behaviors in the Zebrafish Embryo. J. Neurobiol..

[28] González-Fraga J., Dipp-Alvarez V., Bardullas U. (2019). Quantification of Spontaneous Tail Movement in Zebrafish Embryos Using a Novel Open-Source MATLAB Application. Zebrafish.

[29] Fisher D.W., Luu P., Agarwal N., Kurz J.E., Chetkovich D.M. (2018). Loss of HCN2 Leads to Delayed Gastrointestinal Motility and Reduced Energy Intake in Mice. PLoS ONE.

[30] (1993). DiFrancesco, Dario Pacemaker Mechanisms in Cardiac Tissue. Annu. Rev. Physiol..

[31] Moosmang S., Stieber J., Zong X., Biel M., Hofmann F., Ludwig A. (2001). Cellular Expression and Functional Characterization of Four Hyperpolarization-Activated Pacemaker Channels in Cardiac and Neuronal Tissues: HCN Channel Expression and Characterization. Eur. J. Biochem..

[32] Zindler F., Beedgen F., Brandt D., Steiner M., Stengel D., Baumann L., Braunbeck T. (2019). Analysis of Tail Coiling Activity of Zebrafish (Danio Rerio) Embryos Allows for the Differentiation of Neurotoxicants with Different Modes of Action. Ecotoxicol. Environ. Saf..

[33] Zindler F., Beedgen F., Braunbeck T. (2019). Time-Course of Coiling Activity in Zebrafish (Danio Rerio) Embryos Exposed to Ethanol as an Endpoint for Developmental Neurotoxicity (DNT)—Hidden Potential and Underestimated Challenges. Chemosphere.

[34] Kimmel C.B., Patterson J., Kimmel R.O. (1974). The Development and Behavioral Characteristics of the Startle Response in the Zebra Fish. Dev. Psychobiol..

[35] MacPhail R.C., Brooks J., Hunter D.L., Padnos B., Irons T.D., Padilla S. (2009). Locomotion in Larval Zebrafish: Influence of Time of Day, Lighting and Ethanol. Neurotoxicology.

[36] Padilla S., Hunter D.L., Padnos B., Frady S., MacPhail R.C. (2011). Assessing Locomotor Activity in Larval Zebrafish: Influence of Extrinsic and Intrinsic Variables. Neurotoxicol. Teratol..

[37] Li M., Maljevic S., Phillips A.M., Petrovski S., Hildebrand M.S., Burgess R., Mount T., Zara F., Striano P., Schubert J. (2018). Gain-of-Function *HCN2* Variants in Genetic Epilepsy. Hum. Mutat..

[38] Espinosa-Garcia C., Zeleke H., Rojas A. (2021). Impact of Stress on Epilepsy: Focus on Neuroinflammation—A Mini Review. Int. J. Mol. Sci..

[39] Novakova B., Harris P.R., Ponnusamy A., Reuber M. (2013). The Role of Stress as a Trigger for Epileptic Seizures: A Narrative Review of Evidence from Human and Animal Studies. Epilepsia.

[40] An S.-J., Park S.-K., Hwang I.-K., Kim H.S., Seo M.-O., Suh J.-G., Oh Y.-S., Bae J.C., Won M.H., Kang T.-C. (2003). Altered Corticotropin-Releasing Factor (CRF) Receptor Immunoreactivity in the Gerbil Hippocampal Complex Following Spontaneous Seizure. Neurochem. Int..

[41] Forcelli P.A., Orefice L.L., Heinrichs S.C. (2007). Neural, Endocrine and Electroencephalographic Hyperreactivity to Human Contact: A Diathesis-Stress Model of Seizure Susceptibility in El Mice. Brain Res..

[42] Cheng J., Umschweif G., Leung J., Sagi Y., Greengard P. (2019). HCN2 Channels in Cholinergic Interneurons of Nucleus Accumbens Shell Regulate Depressive Behaviors. Neuron.

[43] DiFrancesco J.C., DiFrancesco D. (2015). Dysfunctional HCN Ion Channels in Neurological Diseases. Front. Cell. Neurosci..

[44] Dini L., Del Lungo M., Resta F., Melchiorre M., Spinelli V., Di Cesare Mannelli L., Ghelardini C., Laurino A., Sartiani L., Coppini R. (2018). Selective Blockade of HCN1/HCN2 Channels as a Potential Pharmacological Strategy against Pain. Front. Pharmacol..

[45] Kim C.S., Johnston D. (2018). A Possible Link between HCN Channels and Depression. Chronic Stress.

[46] Moreno-Mateos M.A., Vejnar C.E., Beaudoin J.-D., Fernandez J.P., Mis E.K., Khokha M.K., Giraldez A.J. (2015). CRISPRscan: Designing Highly Efficient SgRNAs for CRISPR-Cas9 Targeting in Vivo. Nat. Methods.

[47] Vejnar C.E., Moreno-Mateos M.A., Cifuentes D., Bazzini A.A., Giraldez A.J. (2016). Optimized CRISPR–Cas9 System for Genome Editing in Zebrafish. Cold Spring Harb. Protoc..

[48] Meeker N.D., Hutchinson S.A., Ho L., Trede N.S. (2007). Method for Isolation of PCR-Ready Genomic DNA from Zebrafish Tissues. Biotechniques.

